# AntiBP2: improved version of antibacterial peptide prediction

**DOI:** 10.1186/1471-2105-11-S1-S19

**Published:** 2010-01-18

**Authors:** Sneh Lata, Nitish K Mishra, Gajendra PS Raghava

**Affiliations:** 1 Institute of Microbial Technology, Sector 39A, Chandigarh, India

## Abstract

**Background:**

Antibacterial peptides are one of the effecter molecules of innate immune system. Over the last few decades several antibacterial peptides have successfully approved as drug by FDA, which has prompted an interest in these antibacterial peptides. In our recent study we analyzed 999 antibacterial peptides, which were collected from Antibacterial Peptide Database (APD). We have also developed methods to predict and classify these antibacterial peptides using Support Vector Machine (SVM).

**Results:**

During analysis we observed that certain residues are preferred over other in antibacterial peptide, particularly at the N and C terminus. These observation and increased data of antibacterial peptide in APD encouraged us to again develop a new and more robust method for predicting antibacterial peptides in protein from their amino acid sequence or given peptide have antibacterial properties or not. First, the binary patterns of the 15 N terminus residues were used for predicting antibacterial peptide using SVM and achieved accuracy of 85.46% with 0.705 Mathew's Correlation Coefficient (MCC). Then we used the binary pattern of 15 C terminus residues and achieved accuracy of 85.05% with 0.701 MCC, latter on we developed prediction method by combining N & C terminus and achieved an accuracy of 91.64% with 0.831 MCC. Finally we developed SVM based model using amino acid composition of whole peptide and achieved 92.14% accuracy with MCC 0.843. In this study we used five-fold cross validation technique to develop all these models and tested the performance of these models on an independent dataset. We further classify antibacterial peptides according to their sources and achieved an overall accuracy of 98.95%. We further classify antibacterial peptides in their respective family and got a satisfactory result.

**Conclusion:**

Among antibacterial peptides, there is preference for certain residues at N and C terminus, which helps to discriminate them from non-antibacterial peptides. Amino acid composition of antibacterial peptides helps to demarcate them from non-antibacterial peptide and their further classification in source and family. Antibp2 will be helpful in discovering efficacious antibacterial peptide, which we hope will be helpful against antibiotics resistant bacteria. We also developed user friendly web server for the biological community.

## Background

In the past few decades, a large number of bacterial strains have evolved ways to adapt or become resistant to the currently available antibiotic [1]. The widespread resistance of bacterial pathogens to conventional antibiotics has prompted renewed interest in the use of alternative natural microbial inhibitors such as antimicrobial peptides. Antimicrobial peptides (AMPs) are a family of host-defense peptides most of which are gene-encoded and produced by living organisms of all types [2-8]. Antimicrobial peptides (AMPs) are small molecular weight proteins with broad spectrum antimicrobial activity against bacteria, viruses, and fungi [3,10]. These evolutionarily conserved peptides are usually positively charged and have both a hydrophobic and hydrophilic side that enables the molecule to be soluble in aqueous environments yet also enter lipid-rich membranes. Once in a target microbial membrane, the peptide kills target cells through diverse mechanisms [5].

Antimicrobial peptides have a broad spectrum of activity and can act as antibacterial, antifungal, antiviral and sometimes even as anticancer peptide [10]. These antibacterial peptides have other properties like antibacterial activity, mitogen activity or act as signaling molecules including pathogen-lytic activities [10]. Extensive work has been done in the field of antibacterial peptide, describing their identification, characterization, mechanism of action etc. keeping in mind their numerous biotechnological applications [11-13]. Lot of work has been done to collect and compile these peptides in form of a database [14-17].

These antibacterial peptides have very low sequence homology, despite their common function [18]. Previously we developed a very robust method AntiBP [19], for predicting antibacterial peptide using SVM, QM (quantitative matrix) and artificial neural network (ANN). Growth of antibacterial peptides in APD database in the last 2 years motivated us to develop a prediction method based on the newer and larger (almost double) dataset. We once again analyzed the antibacterial peptides and developed SVM based models to predict antibacterial peptides, because our previous study show that SVM over perform than other method. In AntiBP2 we also extracted clean dataset of antibacterial peptide families from Swiss-Prot and developed classification models for them. In the following text, we first discuss the method developed to distinguish antibacterial peptides from non-antibacterial peptides (prediction part) and in the next step describe the method for classifying these peptides on the basis of source and classes (classification part).

## Results

### Analysis of the antibacterial peptides

**Figure 1:**
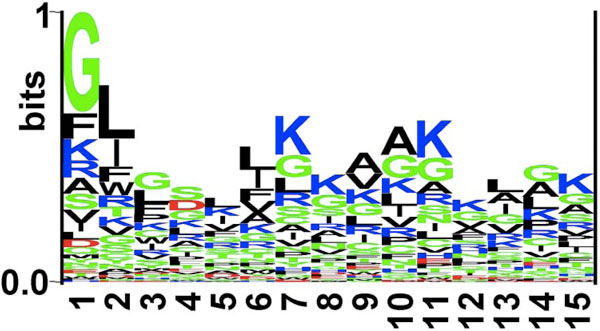
**Sequence logo of first fifteen residues (N-terminus) of antibacterial peptides**. The figure depicts the sequence logo of first fifteen residues (N-terminus) of antibacterial peptides, where size of residue is proportional to its propensity.

**Figure 2:**
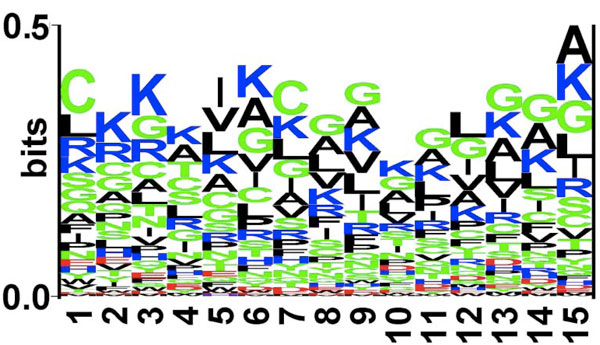
**Sequence logo of last fifteen residues (C-terminus) of antibacterial peptides**. The figure depicts the sequence logo of last fifteen residues (C-terminus) of antibacterial peptides, where size of residue is proportional to its propensity.

Analysis of antibacterial peptides in AntiBP [19] had shown a preference for certain residues over others at both the termini. By drawing the pLOGOs [20] it was also seen that there seems to be a residue preference at different position of antibacterial peptides. As the dataset in AntiBP2 was almost double in size compared to the dataset used in the previous method AntiBP, we again decided to analyze the antibacterial peptides and look for any change or shift in preference trend. We again generated sequence logos of 15 N-terminal and C-terminal residues using pLOGO program (Figures 1 and 2).

**Figure 3:**
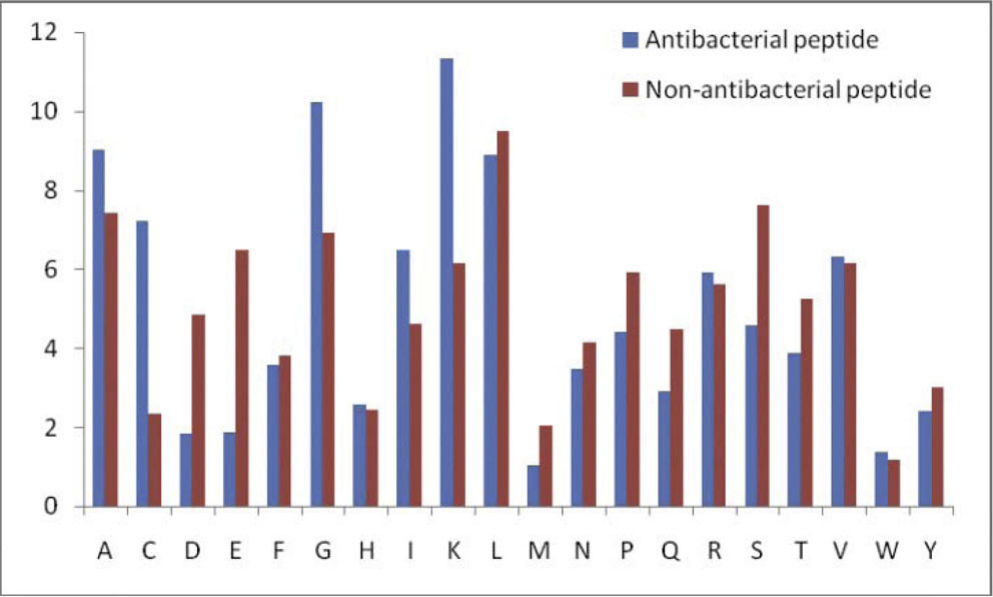
**Overall comparison of 15 N-terminal antibacterial peptide and non-antibacterial**. This figure shows the composition biasness of various amino acids in antibacterial and non-antibacterial peptides at N terminal.

**Figure 4:**
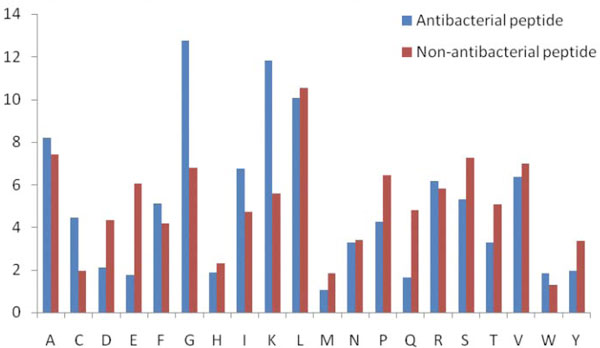
**Overall amino acids comparison of 15 C-terminus antibacterial and non-antibacterial peptides**. This figure shows the composition biasness of various amino acids in antibacterial and non-antibacterial peptides at C terminal.

It was seen that the pLOGOs drawn in AntiBP2 showed similar trend as shown in the method AntiBP [19]. Here also in the N-terminus dataset G, F, V, R was predominating at first position and L, I, W, F were frequently present at 2nd position. Similarly, certain residues are preferred at the C-terminus, for example residues K, G, C, and R are preferred at most of the positions. Though both N and C terminus have a higher proportion of positively charged residues but in AntiBP2 analysis also we could notice a higher frequency of positively charged residues at the C-terminus as compared to the N-terminus (Figures 3 and 4). This may be because it is the C-terminus first interacts with the negatively charged membrane of the bacteria and penetrate it [21]. The N-terminus later helps to hamper the crucial bacterial metabolic functions by interacting with intracellular components like DNA and RNA [22]. Antibacterial peptides also have a high propensity of the residues Cys which is normally not preferred in most of the proteins

**Figure 5:**
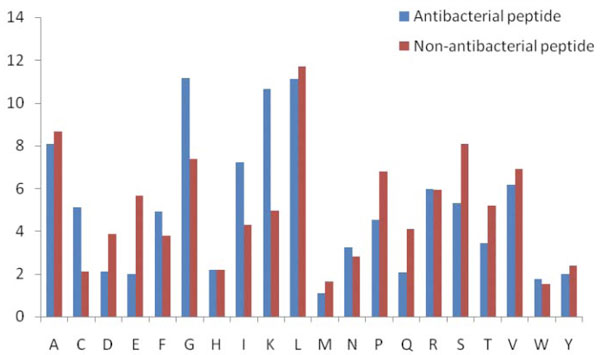
**Overall amino acids comparison of antibacterial peptide and non-antibacterial peptides**. This figure shows the composition biasness of various amino acids in antibacterial and non-antibacterial peptides.

Overall amino acids composition comparison of antibacterial and non-antibacterial shows positively charged Lys is prominent in antibacterial peptides (Figure 5). Similarly Gly and Ile propensity is also high in antibacterial peptides

### Prediction

The performances of NT15, CT15, NTCT15 and whole peptide based prediction method for antibacterial peptides are given below in Table 1. The accuracies achieved by NTCT15 model and whole peptide based model were almost equal (~91%) and is highest among all the models. The performance of NT15 model was better that that of CT15 model.

**Table 1:** Performance of prediction methods developed on NT15, CT15, NTCT15 and whole peptide dataset.

	Sensitivity (%)	Specificity (%)	Accuracy (%)	MCC
**NT15**	84.14	86.77	86.46	0.705
**CT15**	85.5	84.61	85.05	0.701
**NTCT15**	92.22	90.24	91.64	0.831
**Whole peptide**	90.59	93.69	92.14	0.843

### Performance on independent or blind dataset

The prediction models developed in this study were evaluated on a 466 sequence independent dataset (Table 2). These antibacterial peptides in the independent dataset were not used for developing above models either in training or testing.

**Table 2:** Performance of NT15, CT15, NTCT15 and whole peptide dataset model on independent dataset.

	Correctly predicted	Accuracy (%)
**NT15**	361	**77.47**
**CT15**	359	77.04
**NTCT15**	395	84.76
**Whole peptide**	408	87.55

### Classification

The result of classification of antibacterial peptides into 5 sources is given in Table 3. The MCC achieved by the classification model was 0.89, 0.95, 0.94, 0.94 and 0.90 for bacteria, frog, insects, mammals and plants respectively.

**Table 3:** Peformance SVM models in classification of antibacterial peptides according to their source.

	Sensitivity (%)	Specificity (%)	Accuracy (%)	MCC
**Bacteria**	83.33	99.76	97.89	0.89
**Frog**	99.06	96.98	97.68	0.95
**Insects**	93.07	99.65	98.95	0.94
**Mammals**	96.52	97.35	97.05	0.94
**Plants**	90.67	99.2	98.52	0.9

The dateiled results of classification of insect antibacterial peptides into thie listed 5 subfamilies is given in Table 4. For classification of insect antibacterial peptides into Apidaecin, Attacin, Cecropin, Invertebrate defensin and Lebocin, the respective MCC's achieved were 0.92, 0.96, 1, 1, 0.79.

**Table 4:** Classification of insect antibacterial peptides into families.

	Sensitivity (%)	Specificity (%)	Accuracy (%)	MCC
**Apidaecin**	100	98.95	99.01	0.92
**Attacin**	93.75	100	99.01	0.96
**Cecropin**	100	100	100	1
**Invertebrate defensin**	100	100	100	1
**Lebocin**	80	98.96	98.02	0.79

The results of classification of frog's antibacterial peptides and mammalian antibacterial peptides into their respective families (5 each) are given in detail in Table 5 and Table 6. The MCC achieved in classification of frog antibacterial peptides into its respective subfamilies (as listed in datasets section) was 0.95, 0.97, 0.95 0.94, 0.97 and that for mammalian antibacterial peptide families were 0.92, 0.97, 0.97, 1, 0.98.

**Table 5:** Classification of frog antibacterial peptides into families.

	Sensitivity (%)	Specificity (%)	Accuracy (%)	MCC
**Bombinin**	96.72	98.84	98.44	0.95
**Brevinin**	99.31	98.29	98.75	0.97
**Caerin**	100	98.97	99.06	0.95
**Dermaseptin**	90.48	100	98.75	0.94
**Other**	95.24	100	99.38	0.97

**Table 6:** Classification of mammal antibacterial peptides into families.

	Sensitivity (%)	Specificity (%)	Accuracy (%)	MCC
**A-defensin**	88.89	99.65	97.68	0.92
**B-defensin**	100	96.51	98.26	0.97
**Cathelicidin**	98.41	99.29	99.13	0.97
**Hepcidin**	100	100	100	1
**Histatin**	97.06	100	99.71	0.98

## Discussion

A great deal of interest is shown nowadays in antibacterial peptides or the so called "nature's antibiotics", which seem to be promising to overcome the growing problem of antibiotic resistance [23-25]. The design of novel peptides with antimicrobial activities requires the development of methods for narrowing down the candidate peptides so as to enable rational experimentation by wet-lab scientists. Attempts have been made to develop methods and strategies for designing effective antimicrobial peptides [26,27]. AntiBP is one such method meant to discover efficacious antibacterial peptides that we hope could prove to be a boon to combat the dreadful antibiotic resistant bacteria. Enormous growth of antibacterial peptide data in the databases motivated us to develop an improved version of AntiBP using the same strategy. The new version was name AntiBP2.

The N and C terminus sequence logos of AntiBP2 dataset were almost similar to those in the previous method AntiBP. This indicates that though there seems to be an absence of great homology or conservation among antibacterial peptides but the pattern of positional preference of certain residues remains constant. We once again developed the prediction method to classify antibacterial peptide from the non-antibacterial peptide. But this time the method was developed using a training data that was double in size to the one previously used. We developed both whole peptide based compositional models as well as binary pattern based terminus approaches. This time we retained the whole peptide based method also as it becomes difficult to predict peptides that are less than 15 residues in length by the binary pattern based terminal models. In this method also we achieved impressive results with all the above approaches but the best performers were the NTCT15 and whole peptide based prediction models (achieving ~91% accuracy). This was followed by the NT15 based prediction model while the CT15 based model being the poorest performer among all. This trend is just similar to what was seen in AntiBP. The performance evaluation of prediction models on the independent dataset followed the trend shown during development of prediction models (in sync with the trend followed by the AntiBP method). The NTCT15 model performed the best followed by NT15 and CT15 models in respective order.

In AntiBP2 we have also developed models that could classify antibacterial peptides further into families with high accuracy. First we successfully made an attempt to develop classification models that could assign the source of origin to predicted antibacterial peptides. The classification models to classify the antibacterial peptides further into corresponding families were also developed. The results attained in all the classification methods clearly indicate that although the antibacterial peptides do no show a greater conservation or homology, but they become more and more as we go down to the level of a particular family. This is evident from the high accuracies achieved for each family in various classification models. Therefore, AntiBP2 is an efficient method that can predict and classify the antibacterial peptides. We hope that our method would help the wet lab scientists to design improved and efficacious antibacterial peptides in future.

## Conclusion

There is a rapid growth in the field of antibacterial peptide research in response to the demand for novel antibacterial agents. AntiBP2 is one such efficient method that can predict and classify the antibacterial peptides and help to find newer antibacterial peptides more speedily and conveniently. We hope that our method would promote the research to design improved and efficacious antibacterial peptides in future.

## Methods

### Dataset

#### Main dataset

The positive dataset for this method was once again fetched from the antimicrobial peptide database APD [17]. We retrieved a total of 999 unique antibacterial peptides from this database. We used this dataset to build the whole peptide composition based SVM models to predict antibacterial peptides of any length.

#### Negative dataset against whole peptide dataset

As there is no source of experimentally proven non-antibacterial peptides, so we adopted the same strategy that was used to generated the negative dataset in AntiBP. We chose to extract random peptides from proteins belonging to all intracellular locations except from the secretary proteins (because antibacterial peptides are mostly secreted outside the cell). Though some of these randomly selected peptides could be antibacterial in nature but the possibilities are remote. To do this we used the data which was used in MitPred [28]. MitPred dataset had proteins belonging to various intracellular locations (nucleus, cytoplasm, ER, golgi complex, mitochondria). These proteins were then mixed and shuffled thoroughly so that the negative dataset does not have overrepresentation of proteins belonging to any particular location. Now we selected those proteins that were >100 amino acids in length. This was done as many of the antibacterial peptides in the positive dataset having >90 residues in length. Now for peptide in the positive dataset, we calculated its length and cut a random peptide of corresponding length from the negative dataset protein. Thus we got 999 negative peptides in result.

#### NT15, CT15 and NTCT15 datasets

We created NT15 and CT15 datasets by taking first fifteen and last fifteen residues respectively from the antibacterial peptides as done in AntiBP [19]. For NTCT15 dataset we concatenated the CT15 peptides with their corresponding NT15 counterparts. To reduce the redundancy in the positive dataset, duplicates were removed and we were left with 782 NT15, 786 CT15 peptides and 861 NTCT15 peptides.

#### Negative dataset against NT15, CT15 and NTCT15 datasets

The strategy to generate the negative datasets for NT15, CT15 and NTCT15 datasets was the same as used in AntiBP. Once again the dataset having thoroughly mixed and shuffled proteins belonging to various subcellular locations was taken. For NT15 and CT15 negative datasets 15 residues long peptides were cut randomly from this dataset. From these peptides we selected 786 peptides to be used as negative dataset against both, NT15 and CT15 datasets. The negative dataset for NTCT15 dataset was created by extracting 861 random peptides (30 residues in length) from the non-secretary protein dataset.

#### Datasets for Subfamily classification

These datasets for classification of antibacterial peptides were extracted from the protein sequence database Swiss-Prot. These include peptides belonging to bacteria, insects, frogs, mammals and peptides categories into plants. The antibacterial peptides belonging to insects further belonged to 5 families i.e. apidaecin, attacin, cecropins, invertebrate defensins and lebocin. The antibacterial peptides belonging to mammals contained alpha-defensin, beta-defensin, cathelicidin, hepcidin and histatin. Frog antibacterial peptides also had sequences from bombinin, brevinin, caerin, dermaseptin, dermorphin, phylloseptin, pleurain, tryptophillin. As the number of peptides in dermorphin, phylloseptin, pleurain and tryptophillin were very less therefore, these were combined into a single class named as "Other".

#### Independent dataset

We took 466 peptides from the family classification dataset (which was fetched from Swiss-Prot) which were not present in our main dataset (taken from APD database). This dataset was not used either for training or testing the method. These peptides served as the independent dataset for evaluating the performance of the prediction models.

#### Techniques used

As the SVM based technique performed the best in the method AntiBP [19], we therefore exploited SVM to develop the prediction method in this case. In this study, all SVM models have been developed using a freely available program SVM_Light [29]. This program allows users to run SVM using various kernels and parameters. In this study, the accuracy was computed at a cut-off score where sensitivity and specificity are nearly equal.

#### Evaluation of parameters

Five-fold cross-validation technique has been used to evaluate the performance of all the models developed in this study. In five fold cross-validation technique a dataset is randomly divided into five sets, where each set consists of nearly equal number of antibacterial peptides and non antibacterial peptides. Four sets are used for training and the remaining set for testing. This process is repeated five times so that each set is used once for testing. The performance of method is average performance of method on five sets. Following parameters has been used for assessing the performance of a method.

Where, TP and TN are correctly predicted antibacterial peptides and non-antibacterial peptides respectively. FP and FN are wrongly predicted antibacterial peptides and non-antibacterial peptides respectively. Sensitivity *(Sn*) or percent coverage of antibacterial peptide is the percentage of antibacterial peptide predicted as antibacterial peptide; specificity *(Sp*) or percent coverage of non-antibacterial is the percentage of non-antibacterial peptide predicted as non-antibacterial peptide; overall accuracy *(Ac*) is the percentage of correctly predicted antibacterial and non antibacterial. The five fold cross validation technique was used for evaluation of all the three methods.

### Prediction of antibacterial peptides

#### Whole peptide based approach

Though it is seen that the terminus approaches are useful to scan the antibacterial peptide in a larger protein sequence but it becomes difficult of predict peptide which are less than 15 residues. Therefore, a whole peptide based SVM model was also developed in order to predict antibacterial peptides of any length. Amino acid composition of the amino acid residues was fed to train the SVM.

#### NT15, CT15 and NTCT15 approach

Again the binary patterns of NT15, CT15 and NTCT15 datasets were used to develop prediction methods as described in AntiBP. The performance was evaluated using Five-fold cross validation technique.

#### Classification of antibacterial peptides

Multiclass SVM was exploited to develop the classification models and thus models were developed to classify the antibacterial peptides belonging to different sources e.g. Bacteria, Insect, Frog, mammals and plants. N SVMs model were constructed for N-class classification. For antibacterial peptide classification, the number of classes was equal to 5. Five 1-v-r SVMs models were constructed for classification of antibacterial peptides. The *i*th SVM was trained with all the samples of *i*th class labelled positive and all other samples labelled negative. An unknown example was classified into the class that corresponds to the SVM with the highest output score. The results for the family prediction are given in Table 2.

Antibacterial peptides belonging to various sources were further classified into families. Classification models were developed for peptides belonging to insects, frogs and mammals. To classify Insect antibacterial peptides into families 5 1-vs-r SVMs were developed. In a similar way 5 1-vs-r SVM models were developed to classify frog and mammalian antibacterial peptides into their respective families. The detailed results of classification of insect, frog and mammalian peptides are given in results section (Table 3, 4 and 5).

## Availability and requirements

We developed a web server AntiBP2 [30] freely available for predicting and classify antibacterial peptides using models developed in this study. This web server was developed on SUN server (model T-1000) under Solaris environment using PERL programming languages.

## Competing interests

The authors declare that they have no competing interests.

## Authors' contributions

SL created dataset and developed the SVM models and NKM re-checked these models. NKM created the backend web server and the front end user interface. GPSR conceived the project, coordinated it and refined the manuscript drafted by SL and NKM.

## References

[B1] Hancock RE Cationic peptides: effectors in innate immunity and novel antimicrobials Lancet Infect Dis 2001 1 156 164 10.1016/S1473-3099(01)00092-5 11871492

[B2] Nicolas P Mor A Peptide as weapons against microorganisms in the chemical defense system of vertebrates Annu Rev Microbiol 1995 49 277 304 10.1146/annurev.mi.49.100195.001425 8561461

[B3] Epand RM Vogel HJ Discovery of antimicrobial peptides and their mechanism of action Biochim Biophys Acta 1999 1462 11 28 10.1016/S0005-2736(99)00198-4 10590300

[B4] Hancock RE Diamond G The role of cationic antimicrobial peptides in innate host defences Trends Microbiol 2000 8 402 410 10.1016/S0966-842X(00)01823-0 10989307

[B5] Van't Hof W Veeran EC Helmerhorst EJ Amerogen AV Antimicrobial peptides: properties and applicability Biol Chem 2001 382 597 619 10.1515/BC.2001.072 11405223

[B6] Shai Y Mode of action of membrane active antimicrobial peptides Biopolymers 2002 66 236 248 10.1002/bip.10260 12491537

[B7] Brogden KA Ackerman M McCray PB Jr Tack BF Antimicrobial peptides in animals and their role in host defences Int J Antimicrob Agents 2003 22 465 478 10.1016/S0924-8579(03)00180-8 14602364

[B8] Ganz T Defensin: antimicrobial peptides of innate immunity Nat Rev Immunol 2003 3 710 720 10.1038/nri1180 12949495

[B9] Bulet P Stocklin R Menin L Anti-microbial peptides: From invertebrate to vertebrates Immunol Rev 2004 198 169 184 10.1111/j.0105-2896.2004.0124.x 15199962

[B10] Kamysz W Okruj M Lukasiak J Novel properties of antimicrobial peptides Acta Biochim Pol 2003 50 461 469 12833170

[B11] Baker B Zambryski P Staskawicz B Dinesk-Kumar SP Signaling in plant-microbe interactions Science 1997 276 726 733 10.1126/science.276.5313.726 9115193

[B12] Osusky M Zhou G Osuska L Hancock RE Kay WW Mishra S Transgenic plant expressing cationic peptide chimeras exhibit broad-spectrum resistance to phytopathogens Nat Biotechnol 2000 18 1162 1166 10.1038/81145 11062434

[B13] Networks of Centers of Excellence http://www.nce.gc.ca/pubs/reports/9697/ann96-97-71_e.htm

[B14] Brahmchary M Krishnan SPT Koh JLY Khan A Seah SH Tan TW Brusic V Bajic VB ANTIMIC: a database of antimicrobial sequences Nucleic Acids Res 2004 32 Database issue D586 D589 10.1093/nar/gkh077 14681487 PMC308766

[B15] Antimicrobial Sequence database http://www.bbcmunits.it/~tossi/amsdb.html

[B16] Whitmore L Wallace BA The Peptaibol Database: a database for sequences and structure of naturally occurring peptaibols Nucleic Acids Res 2004 32 Database issue D593 D594 10.1093/nar/gkh077 14681489 PMC308811

[B17] Wang Z Wang G APD: the Antimicrobial Peptide Database Nucleic Acid Research 2004 32 D590 D592 10.1093/nar/gkh025 PMC308759 14681488

[B18] Hancock RE Chappel DS Peptide Antibiotics Antimicrb Agents Chenother 1999 43 6 1317 1323 10.1128/aac.43.6.1317 PMC89271 10348745

[B19] Lata Sneh Sharma BK Raghava GP Analysis and prediction of antibacterial peptides BMC Bioinfo 2007 8 263 10.1186/1471-2105-8-263 PMC2041956 17645800

[B20] Schneider D Stephens RM Sequence logos: a new way to display consensus sequences Nucleic Acid Research 1990 18 6067 6100 10.1093/nar/18.20.6097 PMC332411 2172928

[B21] Park CB Kim HS Kim HC Mechanism of actions of the antimicrobial peptides buforin II: buforin kills microorganisms by penetrating the cell membrane and inhibiting cellular functions Biochim Biophys Acta 1998 244 253 257 10.1006/bbrc.1998.8159 9514864

[B22] Yonezawa A Kuwahara J Fujji N Sugiura Y Binding of tachyplesin I to DNA revealed by footprinting analysis: significant contribution of secondary structure to DNA binding and implication for biological action Biochemistry 1992 31 2998 3004 10.1021/bi00126a022 1372516

[B23] Bradshaw JP Cationic antimicrobial peptides: issue for potential clinical use BioDrugs 2003 17 233 240 10.2165/00063030-200317040-00002 12899640

[B24] Hancock RE Patrzycat A Clinical development of cationic antimicrobial peptides: from natural to novel antibiotics Curr Drug Targets Infect Disord 2002 2 79 83 10.2174/1568005024605855 12462155

[B25] Scott MG Hancock RE Cationic antimicrobial peptides and their multifunctional role in the immune system Crit Rev Immunol 2002 20 407 431 11145218

[B26] Frecer V Ho B Ding JL De novo design of potent antimicrobial peptides Antimicrob Agent Chemother 2004 48 3349 3357 10.1128/AAC.48.9.3349-3357.2004 PMC514781 15328096

[B27] Tossi A Tarantino C Romeo D Design of synthetic antimicrobial peptides based on sequence amphipathicity Europ J Biochem 1997 250 549 558 10.1111/j.1432-1033.1997.0549a.x 9428709

[B28] Kumar M Verma R Raghava GP Prediction of mitochondrial proteins using support vector machine and hidden markov model J Biol Chem 2006 281 9 5357 5363 10.1074/jbc.M511061200 16339140

[B29] Joachims T Scholkopf B, Burges C, Smola A Making large-scale SVM Learning Practical Advanced in Kernel methods - support vector learning 1999 Cambridge, MA, London, England: MIIT Press 169 184

[B30] http://www.imtech.res.in/raghava/antibp2/

